# New immunotherapy approaches for colorectal cancer: focusing on CAR-T cell, BiTE, and oncolytic viruses

**DOI:** 10.1186/s12964-023-01430-8

**Published:** 2024-01-19

**Authors:** Amin Kamrani, Hadi Nasiri, Ali Hassanzadeh, Javad Ahmadian Heris, Reza Mohammadinasab, Shahram Sadeghvand, Mohammadreza Sadeghi, Zahra Valedkarimi, Ramin Hosseinzadeh, Navid Shomali, Morteza Akbari

**Affiliations:** 1 Department of Immunology, Faculty of Medicine, Tabriz University of Medical Science, Tabriz, Iran; 2 Immunology Research Center, Tabriz University of Medical Sciences, Tabriz, Iran; 3 Department of Applied Cell Sciences, School of Advanced Technologies in Medicine, Tehran University of Medical Sciences, Tehran, Iran; 4 Department of Allergy and Clinical Immunology, Pediatric Hospital, Tabriz University of Medical Sciences, Tabriz, Iran; 5 Department of History of Medicine, School of Traditional Medicine, Tabriz University of Medical Sciences, Tabriz, Iran; 6 Pediatrics Health Research Center, Tabriz University of Medical Sciences, Tabriz, Iran; 7 Department of Molecular Medicine, Tabriz university of medical science, Tabriz, Iran; 8 Department of Medical Biotechnology, Faculty of Advanced Medical Sciences, Tabriz University of Medical Sciences, Tabriz, Iran; 9 Student Research Committee, Tabriz University of Medical Science, Tabriz, Iran

## Abstract

Colorectal cancer is one of the most common causes of mortality worldwide. There are several potential risk factors responsible for the initiation and progression of colorectal cancer, including age, family history, a history of inflammatory bowel disease, and lifestyle factors such as physical activity and diet. For decades, there has been a vast amount of study on treatment approaches for colorectal cancer, which has led to conventional therapies such as chemotherapy, surgery, etc. Considering the high prevalence and incidence rate, scholars believe there is an urgent need for an alternative, more efficacious treatment with fewer adverse effects than the abovementioned treatments. Immunotherapy has emerged as a potential treatment alternative in a few years and has become one of the fastest-evolving therapeutic methods. Immunotherapy works by activating or enhancing the immune system’s power to identify and attack cancerous cells. This review summarizes the most crucial new immunotherapy methods under investigation for colorectal cancer treatment, including Immune checkpoint inhibitors, CAR-T cell therapy, BiTEs, Tumor-infiltrating lymphocytes, and Oncolytic virus therapy. Furthermore, this study discusses the application of combination therapy, precision medicine, biomarker discovery, overcoming resistance, and immune-related adverse effects.

Video Abstract

**Supplementary Information:**

The online version contains supplementary material available at 10.1186/s12964-023-01430-8.

## Introduction

A type of cancer called colorectal cancer usually develops in the colon or rectum, which are both parts of the large intestine. The rectum and colon remove waste from the body and take in water and nutrients from food [1]. Colorectal cancer develops when normal cells in the colon or rectum experience genetic mutations that make them grow and increase out of control, resulting in a tumor. These tumors may have metastasized (malignant) or not (benign) metastases [2]. Several risk factors, including age, family history, a history of inflammatory bowel disease, and lifestyle factors containing physical activity and diet, are responsible for the onset and progression of colorectal cancer. Changes in bowel habits, rectal hemorrhage, abdominal pain or discomfort, fatigue, and unexplained weight loss may indicate colorectal cancer [3]. Surgical procedures, chemotherapy, targeted therapy, radiation therapy, and immunotherapy are all viable treatment options. Early diagnosis and treatment are crucial for positive outcomes [4]. Immunotherapy is a cancer treatment that activates the immune system to fight cancer. The immune system, which comprises a variety of cells, tissues, and organs, collaborates to protect the body from pathogens, foreign objects, and abnormal cells, such as cancer cells [5]. Immunotherapy works by boosting the ability of the immune system to identify and eliminate cancerous cells. Many immunotherapy approaches, including immune checkpoint inhibitors, target immune cells or cancer cells that suppress the immune system. As a result, the immune system can better identify and destroy cancer cells. CAR-T cell therapy involves genetically modifying a patient's immune cells to target and destroy cancer cells [6]. Tumor infiltration lymphocytes (TILs) are immune cells harvested from a patient's tumor, cultured in vitro, and infused back into the patient to target the malignancy [7]. Utilizing viruses infecting and killing cancer cells while activating the immune system to suppress the cancer is known as oncolytic viral treatment [8].

Several cancers, including melanoma, lung cancer, bladder cancer, and some types of blood cancers, have responded well to immunotherapy treatment. Immunotherapy, though, does not always work on all people, and it might also have unfavorable side effects. It is essential to discuss the potential benefits and hazards of immunotherapy with a physician. This paper discusses the various immunotherapy treatments used to treat colorectal cancer, how they work, and potential benefits and drawbacks. It also provides an overview of immunotherapy as a potential treatment for colorectal cancer. The essay will also compare the efficacy of immunotherapy and conventional therapies in treating colorectal cancer and outline possible future research areas to boost immunotherapy's effectiveness in treating this particular illness. This paper aims to give readers a clear understanding of how immunotherapy can stimulate the immune system to fight colorectal cancer.

## Types of immunotherapies for colorectal cancer

### Checkpoint inhibitors

A kind of immunotherapy known as checkpoint inhibitors targets molecules on immune cells or cancer cells that obstruct the immune response. Checkpoint inhibitors block the PD-1 pathway, which cancer cells use to evade immune system recognition. Pembrolizumab, Dostarlimab, and nivolumab are three checkpoint inhibitors that have received FDA approval for the treatment of specific kinds of colorectal cancer [9]. A signal is sent to immune cells by PD-1 when it attaches to its ligand, PD-L1, on cancer cells, ordering them to cease attacking the cancer cells and go dormant. Checkpoint inhibitors stop this interaction, allowing the immune system to identify and attack cancer cells more easily [10] (Table 1). It has been shown that checkpoint inhibitors are an effective treatment for colorectal cancer, particularly in patients whose tumors have distinct genetic changes or have progressed following prior therapies. Checkpoint inhibitors do not work on all people, and side effects like fatigue, nausea, and skin rashes are possible [11]. It is important to note that checkpoint inhibitors are often used in combination with other medicines, such as chemotherapy or targeted therapy, and are not the first-line treatment for colorectal cancer [12].

**Table 1:** FDA-approved monoclonal antibodies to treat colorectal cancer

Name	Target	Indication
Nivolumab	PD-1	microsatellite instability-high (MSI-H) subgroups of patients with advanced colorectal cancer
Pembrolizumab	PD-1	for subgroups of patients with advanced colorectal cancer who have substantial tumor mutational burden, DNA mismatch repair deficiency (dMMR), or microsatellite instability (MSI-H)
Dostarlimab	PD-1	for specific groups of patients with advanced colorectal cancer who suffer from a DNA mismatch repair (dMMR) deficit
Ipilimumab	CTLA-4	for certain subsets of patients with advanced, high-microsatellite-instability colorectal cancer (MSI-H) (in conjunction with nivolumab)
Bevacizumab	VEGF-A	for subsets of patients with advanced colorectal cancer
Ramucirumab	VEGFR2	for subsets of patients with advanced colorectal cancer
Cetuximab	EGFR	for subsets of patients with advanced, EGFR-positive colorectal cancer
Panitumumab	EGFR	for subsets of patients with advanced, EGFR-positive colorectal cancer

Additionally, research is being done to develop innovative immunotherapy and other treatment combinations for colorectal cancer and to find biomarkers that can predict which individuals will respond best to checkpoint inhibitors [13]. The unregulated mismatch repair (MMR) mechanism, which finds and fixes base mispairs as well as insertions and deletions (indels) that take place during DNA synthesis, is present in about 15% of stage I to III colorectal cancers and 5% of metastatic colorectal cancer (mCRC) [14]. Patients with MSI-H/dMMR mCRC who received treatment with the anti-PD-1 drugs pembrolizumab or nivolumab experienced long-lasting anticancer effects, which led the Food and Drug Administration (FDA) to approve these medicines for use in MSI-H/dMMR CRC patients [15]. As first-line therapy for patients with MSI-H/dMMR mCRC, Diaz et al. establish the long-lasting anticancer efficacy of pembrolizumab monotherapy with higher objective response, longer progression-free survival, and complete response, and fewer treatment-related side events [16]. Temozolomide (TMZ), an alkylating drug, can be used to treat a variety of solid tumors, including gliomas, glioblastomas, neuroendocrine tumors, melanoma, and sarcomas [17, 18]. The development of inactivating mutations in MMR genes, such as MSH6, in MGMT-methylated glioblastomas is linked to TMZ resistance in these tumors. In a study, it was shown as a proof-of-concept that TMZ medication could pharmacologically inactivate genes involved in DNA repair while potentially benefiting patients with methylguanine-DNA-methyltransferase (MGMT)-methylated, RAS-mutant mCRC who are resistant to standard-of-care therapies.

### CAR-T cells

Cancer cells are targeted and eliminated through CAR-T cell therapy by genetically altering a patient's T lymphocytes. The chimeric antigen receptors (CARs) on the CAR-T cells are designed to recognize particular cancer cell epitopes [19]. CAR-T cells interact with cancer cells when they come into touch with them and release cytotoxic chemicals that kill the cancer cells. Acute lymphoblastic leukemia and some types of lymphoma are among the blood malignancies for which CAR-T cell therapy has been approved as a treatment. It hasn't yet been given the go-ahead to be used in this environment, though, as it is still in the early stages of development [20]. The difficulty of CAR-T cells in successfully targeting cancer cells while avoiding normal healthy cells presents one of the challenges in developing CAR-T cell treatment for solid tumors, such as colorectal cancer. There are also worries about the therapy's possible toxicity and adverse effects, involving neurotoxicity and cytokine release syndrome [21]. Despite these challenges, research into the use of CAR-T cell therapy for colorectal cancer is underway, and clinical studies are being conducted to assess the effectiveness and safety of this strategy.

### CAR-T against PD-1 and TREM2

In addition to genetic and epigenetic manipulation, the tumor microenvironment (TME), which is made up of immune checkpoints like programmed LAG-3, death-1 (PD-1)/programmed death-ligand 1 (PD-L1), CTLA4, and TIM3 that infiltrate immunosuppressive myeloid cells and regulatory T cells, has a significant impact on the progression and incidence of CRC [22]; this suppresses endogenous anti-tumor immunity and the effects of CAR-T therapy [23]. Chen et al. integrated bispecific antibodies (BsAb) and CAR-T cells into a single immunotherapeutic platform in their investigation. These findings demonstrated that PD-1-TREM2 scFV was secreted into the TME by CAR-T cells and that this technique had higher anti-tumor efficacy than CAR-T cells that only produce PD-1 single-chain fragment variable (scFv). Therefore, the therapeutic effectiveness of CAR-T cells and checkpoint inhibitors may be enhanced by designing CAR-T cells to release a BsAb targeting the TME [24].

### CAR-T cells and CD166

It's interesting to note that CD6 is a recognized ligand for ALCAM/CD166, a 105 kDa transmembrane glycoprotein belonging to the immunoglobulin superfamily that is present on the cell surface. The CUB-domain-containing protein 1 (CDCP1), CD318, and a type I transmembrane protein were recently discovered to bind to CD6 [25, 26]. The degree of target antigen specificity used in CAR-T cell therapy design determines its effectiveness and safety. The full-length ectodomain sequence of CD6, which binds CD166 via the SCRC3 domain and CD318 via the SCRC1 domain, was used to build CARs for colorectal cancer [27].

### CAR-T cells and Nectin4

The type I transmembrane protein nectin cell adhesion molecule 4 (nectin4) works with cadherin to establish and maintain adhesion junctions and has an extracellular domain comprising three Ig-like domains (V-C–C type). Although it is infrequently expressed in normal adult tissues, nectin4 is widely expressed in human embryonic cells and abundantly expressed on the surfaces of malignant solid tumors [28]. Delivering Nectin4-7.19 CAR-T therapy for Nectin4-positive malignant solid tumors may be a practical approach. Co-targeting Nectin4-positive tumor cells and FAP-positive CAFs using Nectin4-7.19 CAR-T cell treatment and FAP-12 CAR-T cell therapy will be a promising synergistic strategy [29].

### CAR-T cells and CEA

A sensitive tumor biomarker for gastrointestinal cancer, carcinoembryonic antigen (CEA) is highly expressed in the tissue and serum of people with colorectal cancer (CRC). Furthermore, immune cells are unable to express CEA in most normal adult tissues, except for the gastrointestinal system, where it is present at a low level and is limited to the apical surface of the epithelial cell membranes facing the lumen [30]—persistence of CAR-T cells in individuals receiving large dosages of CAR-T therapy's peripheral blood. We noted CAR-T cell growth, which was significant, particularly in patients who had received a second CAR-T therapy. Together, we showed that CEA CAR-T cell therapy, even at large doses, was well tolerated in CEA + CRC patients and that most patients who received it showed some efficacy [31].

### CAR-T cells and GUCY2C

Guanylyl cyclase C (GUCY2C) is a membrane-bound receptor that, upon activation by the hormone ligands guanylin or uroguanylin, produces the second messenger, cGMP, controlling intestinal homeostasis, cancer, and obesity [32]. In a syngeneic animal model, GUCY2C CAR-T cells offered long-term protection against lung metastases of murine colorectal cancer cells modified to express human GUCY2C. In a human xenograft model in immunodeficient mice, GUCY2C murine CAR-T cells recognized and destroyed human colorectal cancer cells endogenously expressing GUCY2C. As a result, they have discovered a CAR-T cell therapy strategy that is human GUCY2C-specific and may be created to treat mCRC that represents GUCY2C [33].

### CAR-T cells and CD318

A transmembrane protein known as CD318 or CUB domain-containing protein 1 (CDCP1) has been linked to the loss of the tumor suppressor gene PTEN and the onset of mCRC [34]. In conclusion, it has been suggested that CD318 is a potential CAR-T therapy target for treating CRC. We established its feasibility and efficacy after verifying its anticancer capacity in vitro and in vivo, offering a fresh approach to treating colorectal cancer in the future [35].

### CAR-T cells and other targets

**Fig. 1:**
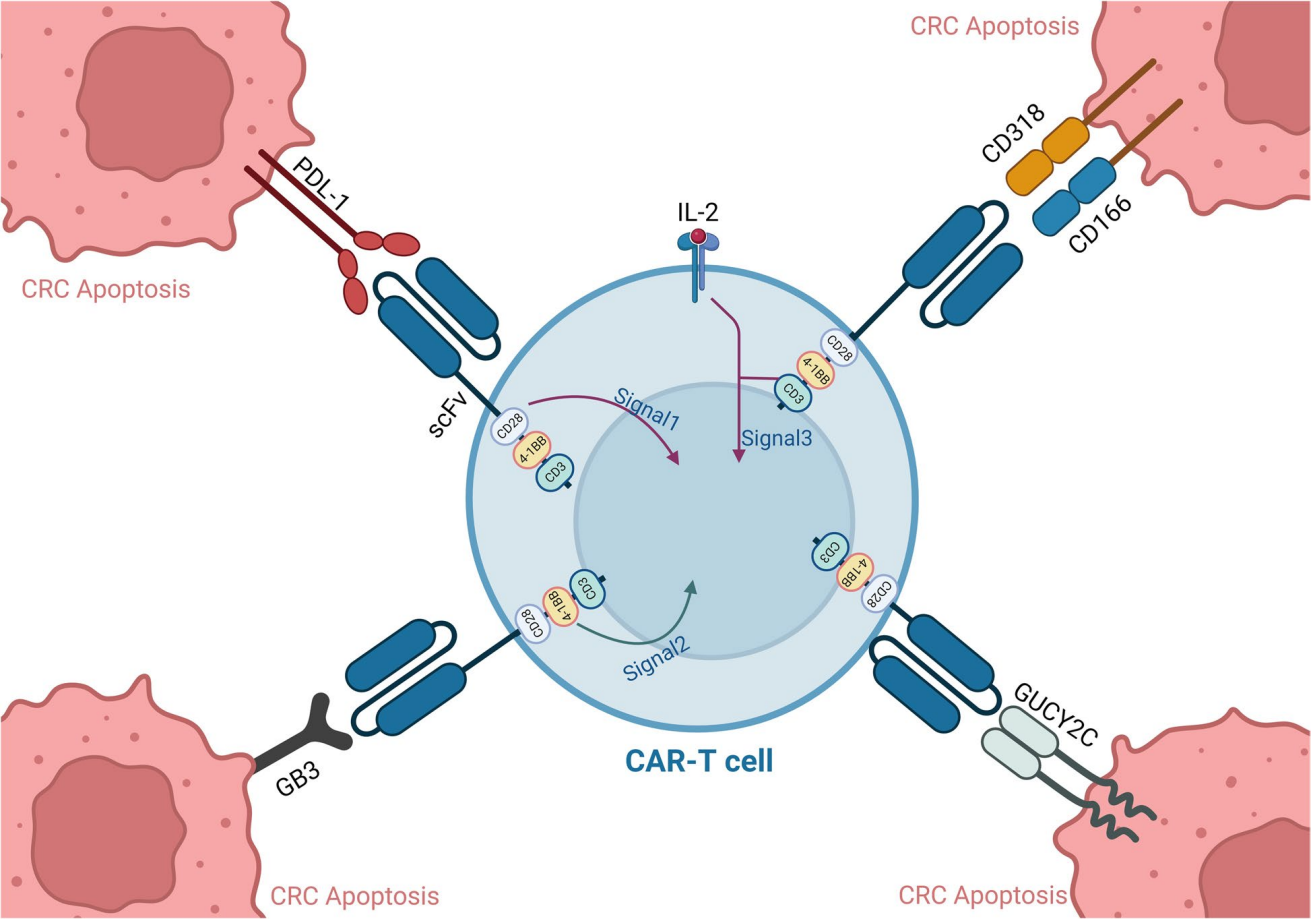
New CAR-T cell strategies, such as specific CAR-T cells against PDL-1, CD166/CD318, GB3, and GUCY2C, can be new immunotherapeutic approaches for CRC apoptosis

The globotriaosylceramide (Gb3), a member of the globo-series and also known as CD77 or Pk antigen, was the first to be recognized as a fibrosarcoma-associated antigen [36]. In addition to gliomas and acute non-lymphocytic leukemia (ANLL), ovary, Burkitt's lymphoma, breast, colorectal carcinoma, and pancreatic cancer all have significant levels of Gb3 expression. Invasiveness, angiogenesis, metastasis, and multidrug resistance have also all been linked to Gb3 [37]. The Gb3-binding lectin-CARs have shown target-specific cytotoxicity against solid tumor cells obtained from colorectal and Burkitt's lymphoma cell lines. These results highlight the enormous therapeutic potential of lectin-based CARs to target Gb3 and other TACAs expressed in solid tumors and hematological malignancies [38] (Table 2) (Fig. 1).

**Table 2:** Recent products in CAR-T cell and BiTE for colorectal cancer

Target	Study Model	Function	Reference
PD-1-TREM2	C57BL/6 mice	Targeting TME and enhancing anti-PD-1 immunotherapy	[24]
CD166	In vitro	Potent cytotoxicity targeting CRC	[27]
Nectin4/FAP	Mice	Suppressed metastatic tumors and increased survival	[29]
CEA	Phase1	Cytokine excretion and cytotoxicities to CEA + CRC	[31]
GUCY2C	Mice	Enhanced antigen-dependent T-cell activation and cytokine production	[33]
CD318	Mice	Activated and exhibited strong cytotoxicity and cytokine-secreting	[35]
Gb3	In vitro	Target-specific cytotoxicity against CRC cells	[38]
EGFR	Mice	lysis of KRAS- and BRAF-mutated CRC lines	[39]
EpCAM	Mice	Blocked xenograft tumor growth	[40]
CEA/BiTE	BALB/c mice	Targeting CEA + Tumor cells	[41]
Cd1d/BiTE	C57BL/6J (B6) mice	Activated invariant natural killer T cells (iNKT cells)	[42]

### bispecific antibodies ScFv (BITE)

**Fig. 2:**
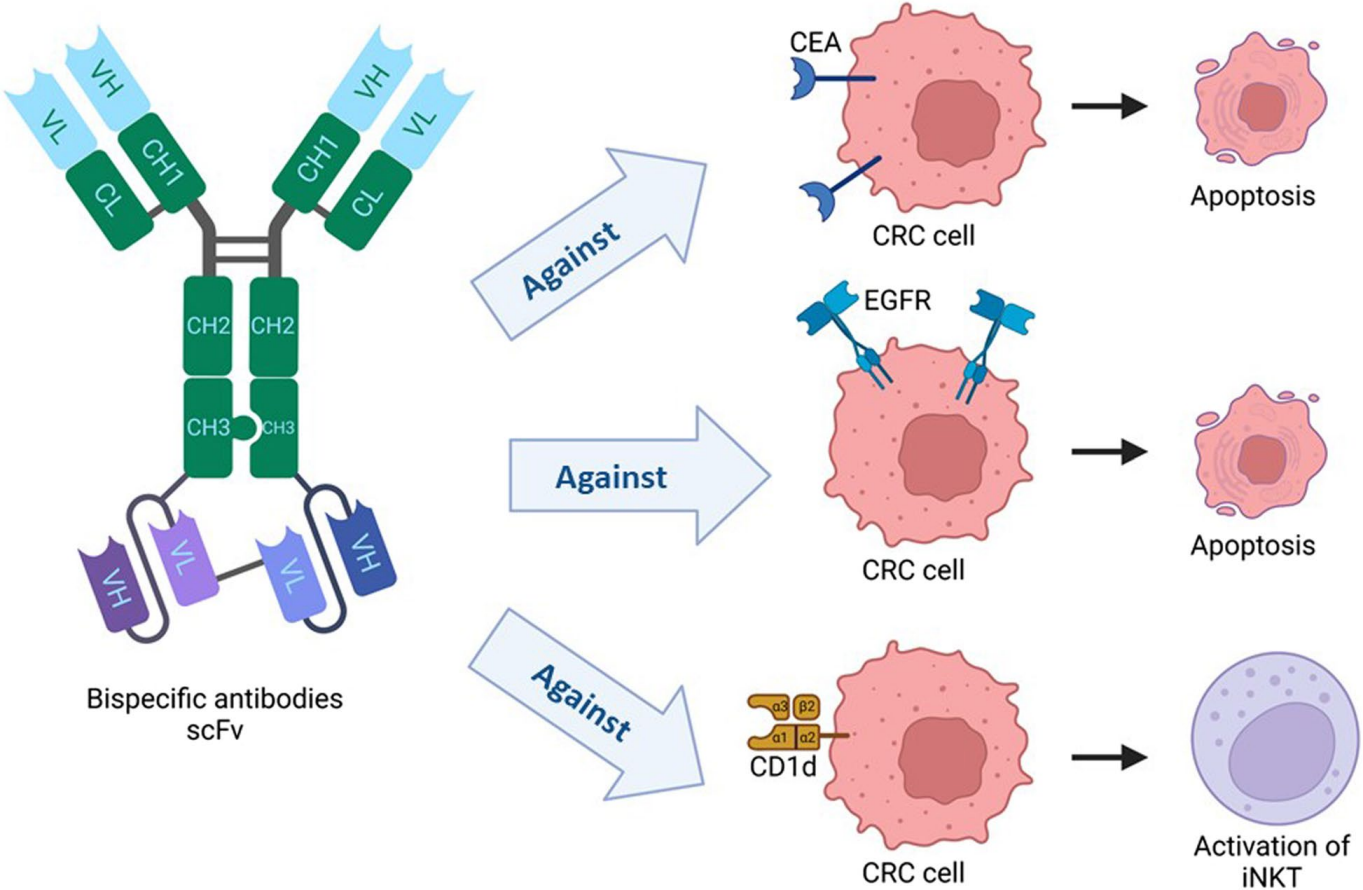
Bispecific antibodies (BiTE) comprise two flexibly linked single-chain antibodies, one binding to the surface antigen on the target cell and the other to CD3 on T cells. Some new BiTEs against CEA, EGFR, and Cd1d can be immunotherapy strategies

In order to start redirected target cell lysis, BiTE antibodies are made to link T cells with cancer cells [43] momentarily. Regular IgG1 antibodies cannot connect with T cells because they lack the Fc receptors required for antibody interaction. BiTE antibodies comprise two flexibly linked single-chain antibodies, one binding to the surface antigen on the target cell and the other to CD3 on T cells. Numerous studies have characterized anticancer activity in cell culture, several xenograft models, and the method of action of BiTE antibodies [43] (Fig. 2). Monoclonal antibodies that target the epidermal growth factor receptor (EGFR) primarily stop the growth of CRC by obstructing receptor signaling. Recent studies have demonstrated that such antibodies do not benefit CRC patients with mutant KRAS and BRAF oncogenes [44]. So, in a study, T cell-engaging BiTE antibodies have been created using the binding domains of cetuximab and panitumumab. Human T cells achieved potent redirected lysis of KRAS- and BRAF-mutated CRC lines at subpicomolar concentrations using both EGFR-specific BiTE antibodies. The growth of tumors from KRAS- and BRAF-mutated human CRC xenografts was likewise inhibited at very low dosages by the cetuximab-based BiTE antibody. At the same time, cetuximab was ineffective in this regard [39].

EpCAM (CD326), a human cell surface glycoprotein that is 39–40 kDa in size and widely expressed in a variety of cancer cells, including ovarian, breast, colorectal, prostate, lung, and pancreatic, is one of the more alluring targets for immunotherapy. EpCAM is engaged in cell proliferation, adhesion, differentiation, survival signaling, and metastasis, in addition to playing a significant role in colorectal cancer biology [45]. In a study, Vita et al. [40] show how to use mRNA-lipid nanoparticle (LNP) technology to deliver bispecific antibodies. EpCAM-CD3 human Fc (hFc) mRNA-LNPs were delivered into mice tumors via intravenous T cell injection, which significantly inhibited the growth of the CRC xenograft tumor in vivo. AMG 211 is a potential novel BiTE antibody design that may be interesting for solid tumors that overexpress CEA. Explants of mCRC cells from patients who progressed on treatment are lysed by AMG 211 in vitro [46].

Additionally, the combination of AMG 211 with immune checkpoint inhibition increased the cytotoxicity of CEA-positive tumor cells in vitro [47]. In live tumor tissue, ^89^Zr-AMG211 demonstrated dose-dependent CEA-specific tumor targeting and localization [41]. A conserved subgroup of specialized T cells known as invariant natural killer T (iNKT) cells is involved in various innate and adaptive immune responses. iNKT cells, which differ from normal T cells in that they carry T cell antigen receptors (TCRs) of restricted diversity, react to particular foreign and self-glycolipid antigens presented by the CD1d protein, which is similar to the MHC class I protein [48]. By disseminating epitopes for tumor-specific CD8 + cytolytic T cell responses, conjugated BiTEs promoted several iNKT cell effector activities, such as cytokine release, secondary activation of NK cells, induction of dendritic cell maturation, and dendritic cell maturation. Combination immunotherapy may be possible due to the simultaneous checkpoint blockade with antibodies to CTLA-4 and the anti-tumor effects of iNKT cell activation with conjugated BiTEs [42] (Table 2).

### Tumor-infiltrating lymphocytes (TILs)

TILs are immunotherapy that fights cancer by taking immune cells out of a patient's tumor, boosting them in a lab, and then reinfusing them into the patient. Utilizing the patient's immune system to fight cancer cells is the aim of TIL treatment. TIL therapy has been investigated for the treatment of numerous cancer types, including melanoma and lung cancer; clinical trials are still ongoing for colorectal cancer [49]. TIL treatment has been demonstrated to have long-lasting effects and even cure some individuals with metastatic melanoma. The fact that there are fewer TILs in colorectal tumors than in other forms of cancer makes it more challenging to collect and multiply enough TILs for therapy, which is one of the challenges of TIL therapy for colorectal cancer [50].

Furthermore, TIL treatment calls for the surgical removal of the tumor, which not all patients may be able to undergo. Despite these challenges, studies are still being conducted in clinical settings to assess the safety and effectiveness of TIL treatment for colorectal cancer. The lymphocytic infiltrates were evaluated based on previously published anatomopathological studies. The inter-observer variability reduces the uniformity of the evaluation. Despite these disadvantages, inflammatory infiltrates in TME have a significant benefit, emphasizing the importance of regularly revealing this feature for colon cancer surgical specimens in clinical treatment [51].

### Oncolytic virus therapy

A type of immunotherapy known as "oncolytic virus therapy" uses engineered viruses to target and destroy cancer cells specifically. The virus is designed to reproduce inside cancer cells, killing them but sparing healthy cells. Because it can target both primary and metastatic tumors, oncolytic viral therapy is a potential treatment for colorectal cancer [52] (Table 3). Clinical trials have demonstrated promise for the oncolytic virus talimogene laherparepvec (T-VEC), which has been approved for the treatment of metastatic melanoma. Early-phase clinical trials and preclinical models have been used to study oncolytic viral therapy for colorectal cancer. According to studies, the oncolytic virus can improve the effectiveness of other therapies like chemotherapy and immunotherapy and induce an immune response against cancer cells [53]. Oncolytic viral therapy for colorectal cancer is still being investigated for its efficacy, and more work is needed to establish the ideal dosage and course of treatment and find the biomarkers that can predict which patients would respond to the therapy the best. Inflammation and damage to healthy tissues are among the potential side effects of oncolytic viral therapy that are of concern [54]. It is crucial to note that not all immunotherapies are effective for all patients with colorectal cancer, and there is still a great deal of research needed to determine the most effective treatments for the various types and stages of colorectal cancer.

**Table 3:** Different oncolytic viruses’ immunotherapy for CRC

Type of oncolytic viruses	Targets	Function	References
Bispecific Engager Viruses	CEACAM5	Decreasing metastasis of CRC cells	[55]
Pseudorabies virus	HCT-8	Suppressed tumor proliferation both in vitro and in vivo	[56]
PexaVec	PD-1/CTLA-4	mismatch repair proficient (pMMR) metastatic CRC	[57]
Adenovirus H101	PD-1	promotes intratumor T-cell infiltration	[58]
Human adenovirus type 5	CRC specific Ag	Decreases patient complications and improves the patient’s quality of life	[59]
**VAX014**	Ki67	Decreased Ki67 proliferation	[60]
**HSV-1**	-	Increased IL-12 and CXCL-11	[61]

### Recent studies in colorectal cancer and oncolytic viruses therapy

CXCL10-CXCR3 signaling is essential for T-cell tumor invasion and tumor immunotherapy. Oncolytic viruses (OVs) induce tumor invasion and effective T-cell immunity. One example of an OV is an oncolytic adenovirus (AdV). Consequently, equipping OV with CXCL10 might be a tempting tactic to get around resistance to anti-PD1 therapy [62]. Adv-CXCL10 is a brand-new oncolytic recombinant adenovirus that encodes murine CXCL10. Through intratumoral injection, the functional chemokine CXCL10 is continuously expressed in the TME, attracting more CXCR3 + T cells there to kill tumor cells, and the recombinant adenovirus exhibits excellent ability to 'fire up' the TME and improve the anti-tumor efficiency of PD-1 antibodies [63]. Using intravenous delivery of our oncolytic virus-driven T-cell-based combination immunotherapy to target colorectal tumors and CTLA4-positive Treg cells in the microenvironment of the cancer, it has been seen that lung metastases of colorectal tumors have significantly decreased [55].

In conclusion, we developed a unique combination method for treating colorectal malignancies by employing the oncolytic vaccinia virus to improve immune-payload delivery and heighten T-cell responses within tumors. PRV, a significant pig viral pathogen, is a member of the *Alphaherpesvirinae* subfamily of the Herpesviridae family. It is a considerable pig viral pathogen. It can result in reproductive issues for sows, respiratory conditions, and growth retardation in fattening pigs, and problems of the nervous system in early piglets [64]. In conclusion, the PRV gene-deleted vaccination strain prevented the development of BALB/c nu mouse xenograft tumor models in vivo and CRC cells in vitro. The PRV HB98 strain in CRC cells demonstrated a more substantial anti-tumor impact and was less harmful than the Bartha K61 strain. In CRC cells and tumor models created from xenografted BALB/c mice, the oncolytic PRV may cause apoptosis. The oncolytic virus pexastimogene devacirepvec (PexaVec, JX-594) was genetically altered [56, 65].

By tumor cell death and the consequent release of tumor antigens, pexaVec therapy creates a proinflammatory tumor environment ideal for immunotherapy [66]. Studies with immunomodulatory therapies show a high association between immunotherapy targets like PD-L1 and tumor-infiltrating T cells in response to this treatment combination [67]. It is safe and tolerable to use PexaVec together with durvalumab and tremelimumab. No unforeseen toxicities were found, and PexaVec, durvalumab, and tremelimumab showed potential therapeutic activity in people with mismatch repair proficient (pMMR) metastatic CRC [57]. The therapeutic effectiveness of PD1 mAb was improved by increased tumor-associated CD8 + cytotoxic T-cell infiltration and higher PD-L1 levels. Therefore, a promising approach to anti-tumor therapy is the combination of oncolytic virus and tumor immune checkpoint blocking (ICBs) [68]. In a mouse model of CRC, treatment with an oncolytic adenovirus successfully inhibits tumor growth. Combinatorial therapies with an oncolytic adenovirus and PD-1 mAb therapy, in this case, demonstrated a high immunotherapeutic efficacy with excellent safety; in addition, it enhances the anti-tumor response in CRC, providing a promising approach to treating patients with CRC [58]. For the metastasis of gastric and colorectal cancer, the liver is a crucial target organ. The management of liver metastases is one of the difficulties in the treatment of gastric and colorectal cancer. The effectiveness, side effects, and coping mechanisms of oncolytic virus injection in patients with liver metastases of gastrointestinal malignancies have been examined in a study. Recombinant human adenovirus type 5 can be successfully treated in patients with liver metastases of gastrointestinal malignant tumors using nursing-based interventions; this is crucial for clinical therapy since it considerably lowers patient problems and raises the standard of living for patients [59].

In the clinical stage, an oncolytic recombinant bacterial minicell (rBMC) therapy called VAX014 is made from *Escherichia coli* cells that include immune-attenuated lipopolysaccharide (LPS). Invasion, which naturally targets unligated alpha3beta1 and alpha5beta1 integrin heterodimers on tumor cells, is the mechanism by which VAX014 is tumor-specific. In non-muscle invasive bladder cancer preclinical models, VAX014 decreased tumor growth and increased survival [69]. A decrease in Ki67 proliferation in vivo following neoadjuvant VAX014 treatment may indicate that VAX014 suppresses the growth of adenomas via both oncolytic and immunotherapeutic effects. When taken together, these studies show promise for VAX014 treatment in populations with CRC and "at risk" polyp-bearing or early adenocarcinoma [60]. Nearly 90% of people have or are carriers of the highly contagious human disease herpes simplex virus type 1 (HSV-1). The 152-kb linear double-stranded DNA of HSV-1 contains about 84 open reading frames organized for contiguous transcription [70]. Several HSV-1 genes are crucial in preventing host immunological reactions. For example, the neurovirulent factor ICP34.5 targets the stimulator of interferon genes (STING), which causes the interferon regulatory factor 3 (IRF3) to be downregulated and type I interferon to be produced [71]. The HSV-1 viral genome was successfully modified for oncolytic viruses using the CRISPR/Cas9 system. Additionally, according to Zhang et al. [61], the dual gene (IL12 and CXCL11)-armed oncolytic virus can work in concert to enhance the effectiveness of colon cancer treatments. The straightforward and affordable application of the CRISPR/Cas9 system enables future research into various immunostimulatory cytokines and oncolytic viruses containing three or more genes as possible cancer therapeutics.

### Future directions for immunotherapy for colorectal cancer

Several promising avenues for future research and development in colorectal cancer immunotherapy are evolving swiftly. Here are some of the most critical areas of future immunotherapy research for colorectal cancer:

### Combination therapies

Combination therapy entails using multiple drugs or treatments to target cancer cells in various ways to improve treatment outcomes. Combining multiple types of immunotherapy or immunotherapy with traditional cancer treatments such as chemotherapy or radiation therapy may increase treatment efficacy and response rates in the context of immunotherapy for colorectal cancer [72]. Several combination therapies for colorectal cancer have been investigated. Combining checkpoint inhibitors, such as anti-CTLA-4 and anti-PD-1 antibodies, has increased response rates and overall survival in colorectal cancer patients [73]. Combining checkpoint inhibitors with chemotherapy has also demonstrated promise for enhancing the prognosis of colorectal cancer patients. Combining checkpoint inhibitors with targeted therapy: Combining checkpoint inhibitors with targeted therapy drugs, such as anti-EGFR or anti-VEGF inhibitors, can enhance the prognosis for some patients [74]. Combining immunotherapy and radiation therapy: According to preclinical research, combining immunotherapy and radiation therapy can boost the immune response and improve the prognosis for patients with colorectal cancer [75]. Combining immunotherapy with CAR-T cell therapy may be a promising treatment option for patients with colorectal cancer who do not respond to standard treatments. The safety and efficacy of various combinations of immunotherapy and other treatments for colorectal cancer are currently being investigated in ongoing clinical trials. The outcomes of these investigations may provide patients with this disease with new treatment options and improve their prognoses.

### New approaches to overcoming resistance

Treatment of colorectal cancer must overcome immunotherapy resistance. However, scientists are continuously looking at novel solutions to this problem. Here are several fresh strategies that show promise: combining treatments Combination medicines, as previously mentioned, may be successful in overcoming immunotherapy resistance. Combining immunotherapy with chemotherapy or targeted therapy may improve some patients' response rates and overall survival (OS). Bispecific antibodies are synthetic proteins that can cling to two targets simultaneously. In the context of immunotherapy for colorectal cancer, bispecific antibodies can be created to target cancer cells and immune cells, ignoring immunotherapy resistance [76]. Immune checkpoint agonists: Immune checkpoint agonists increase signals that can enhance the immune response, whereas immune checkpoint inhibitors block signals that prevent immune cells from attacking cancer cells [77].

Preclinical research suggests that immunotherapy resistance may be overcome in some patients by immune checkpoint agonists [78]. Adoptive T cell transfer involves harvesting T cells from a patient and genetically modifying them to target tumors more effectively. The modified T cells are then reinfused into the patient to stimulate the immune system. This strategy has shown promise in overcoming immunotherapy resistance in colorectal cancer patients [79]. Immunomodulatory pharmaceuticals are intended to modify the immune system in various ways. Some drugs, for instance, can increase the activity of immune cells or obstruct signals that prevent immune cells from attacking cancer cells. Some patients may be able to overcome immunotherapy resistance with the help of these medications. New strategies for circumventing immunotherapy resistance are an active area of study. Ongoing clinical trials are examining the safety and efficacy of these new approaches, and the results of these studies may provide new treatment options for colorectal cancer patients who do not respond to current therapies [80].

## Conclusion

Colorectal cancer is a type of cancer that affects the colon or rectum. Immunotherapy is a cancer treatment that uses the immune system's power to fight cancer cells. Several immunotherapies can be used to treat colorectal cancer, including checkpoint inhibitors, CAR-T cells, tumor-infiltrating lymphocytes, and oncolytic virus therapy. Immunotherapy is effective because it enhances the immune system's capacity to recognize and destroy cancer cells. Immunotherapy has shown promise in increasing response rates and overall survival for some colorectal cancer patients, but it is not practical for all. Resistance to immunotherapy is a significant issue in treating colorectal cancer. Scientists are actively researching new methods to overcome this problem, including combination therapies, bispecific antibodies, immune checkpoint agonists, adoptive T cell transfer, and immunomodulatory drugs; one of the main benefits of immunotherapy which can be especially significant for patients with advanced colorectal cancer that has spread to other organs. Patients who have not responded to conventional treatments may also benefit from immunotherapy. For instance, some patients with advanced colorectal cancer may have tumors that cannot be surgically removed or tumors that have developed chemotherapy resistance. Genetically modifying a patient's T lymphocytes targets and eradicates cancer cells with CAR-T cell therapy. Research is ongoing to determine the appropriate dosage and course of treatment for oncolytic viral therapy for colorectal cancer and identify the biomarkers to help identify which patients will respond most well to this treatment. Overall, immunotherapy's prospective impact on colorectal cancer treatment is significant. As researchers continue investigating new strategies for overcoming immunotherapy resistance and enhancing response rates, colorectal cancer patients may experience even more essential benefits.

## Abbreviations

TILs: Tumor infiltration lymphocytes
mCRC: Metastatic colorectal cancer
CARs: Chimeric Antigen Receptors
TME: Tumor Microenvironment
PD-L1: Programmed Death-Ligand 1
T-VEC: Oncolytic virus talimogene laherparepvec
CTLA4: Cytotoxic T-lymphocyte–associated antigen 4
MSI-H: Microsatellite instability
dMMR: Deficient mismatch repair failure
CDCP1: CUB domain-containing protein 1
ANLL: Acute non-lymphocytic leukemia
EGFR: Epidermal growth factor receptor
iNKT: Invariant natural killer T
TCRs: T cell antigen receptors
OVs: Oncolytic viruses
AdVs: Oncolytic adenoviruses
CXCL10: C-X-C motif chemokine 10
ICB: Immune checkpoint blocking
